# Progress in cultivation-independent phyllosphere microbiology

**DOI:** 10.1111/1574-6941.12198

**Published:** 2013-09-30

**Authors:** Thomas Müller, Silke Ruppel

**Affiliations:** 1 Leibniz-Centre for Agricultural Landscape Research, Müncheberg, Institute of Landscape BiogeochemistryMüncheberg, Germany; 2 Leibniz-Institute of Vegetable and Ornamental CropsGroßbeeren/Erfurt, Germany

**Keywords:** nonculturability, microbial genetic and metabolic diversity, interactions between plants and microorganisms, enteric human pathogens

## Abstract

Most microorganisms of the phyllosphere are nonculturable in commonly used media and culture conditions, as are those in other natural environments. This review queries the reasons for their ‘noncultivability’ and assesses developments in phyllospere microbiology that have been achieved cultivation independently over the last 4 years. Analyses of total microbial communities have revealed a comprehensive microbial diversity. 16S rRNA gene amplicon sequencing and metagenomic sequencing were applied to investigate plant species, location and season as variables affecting the composition of these communities. In continuation to culture-based enzymatic and metabolic studies with individual isolates, metaproteogenomic approaches reveal a great potential to study the physiology of microbial communities *in situ*. Culture-independent microbiological technologies as well advances in plant genetics and biochemistry provide methodological preconditions for exploring the interactions between plants and their microbiome in the phyllosphere. Improving and combining cultivation and culture-independent techniques can contribute to a better understanding of the phyllosphere ecology. This is essential, for example, to avoid human–pathogenic bacteria in plant food.

## Introduction

The phyllosphere is the surface and interior of the aerial parts of vascular plants (Newton *et al*., 2010). It is colonized by bacteria, filamentous fungi, yeasts, archaea and protists that have adapted to life under nutrient and water resource limitations, UV exposure, high temperature shifts and the presence of reactive oxygen species (Lindow & Brandl, 2003; Newton *et al*., 2010; Knief *et al*., 2011). Bacteria are the most common microorganisms in this habitat, and therefore, they are the focus of most studies. The interest in phyllosphere microbiology was initially driven by investigations into plant pathogens. However, most phyllosphere-colonizing microorganisms live as commensals on their host plants. The lives and the ecology of these nonpathogens are much less understood. Currently, it is largely unknown to what extent plants benefit from commensal microbiota colonizing their above-ground surfaces (Innerebner *et al*., 2011; Knief *et al*., 2011). They are thought to play a crucial role in the cycling of elements as saprophytes and in remediating residual pesticides and atmospheric hydrocarbon pollutants, or they may be of relevance for plant development and health as biofertilizers, phytostimulators and biopesticides to protect against invading pathogens (Lugtenberg *et al*., 2002; Delmotte *et al*., 2009; Zhou *et al*., 2011; Ali *et al*., 2012).

Whipps *et al*. (2008) published a comprehensive review of phyllosphere microbiology with special reference to diversity and plant genotypes. The authors also recommended future directions for phyllosphere research, namely the functional consequences of changes in community structures and the mechanisms by which plants control the microbial populations on their aerial plant surfaces.

In the most recent review of microbial ecology in the phyllosphere, Vorholt (2012) highlighted fundamental studies elucidating conserved mechanisms through which microorganisms survive on above-ground plant parts. Improving our understanding of the behaviour of microorganisms in this habitat should facilitate biotechnological applications for protecting plants, promoting plant growth and avoiding human pathogenic bacteria in plant food and the phytoremediation of volatile pollutants from the air.

Specialized reviews have referred to phyllosphere microorganisms in crop protection (Newton *et al*., 2010) and plant–bacterial interactions in soya bean and rice (Ikeda *et al*., 2010a). Much attention has been paid to the persistence and distribution of enteric human pathogenic bacteria on fruit and vegetables in recent years. The ecology as well as the interactions between these bacteria and the plant-associated microbiota has been addressed in reviews by Critzer & Doyle (2010), Teplitski *et al*. (2011) and Brandl *et al*. (2013).

The realization that a substantial portion of the microorganisms associated with plants, such as those in other natural environments too, is viable or metabolically active, but nonculturable in commonly used media and culture conditions, has had important implications for plant microbiology and has brought about the introduction of culture-independent detection methods into phyllosphere research (Wilson & Lindow, 2000). Procedures used in the last decade to analyse the composition of microbial communities in leaf samples without any bias of cultivation have been mainly based on the 16S rRNA gene amplification and amplicon sequencing. This technology has the advantage of assessing a broader spectrum of microbial colonizers than culture techniques; however, it comprises the weaknesses of PCR amplification, such as sensitivity to inhibitory compounds, primer mismatch sensitivity, lack of quantitative information, and primarily, the amplification of interfering plant organelle-derived RNA sequences (Saito *et al*., 2007; Berlec, 2012). Whole metagenome shotgun sequencing of DNA extracted from complex microbial communities in environmental samples enables high-throughput genome analyses, which result in metagenomes that provide information on individual genes or organisms in a particular ecosystem (Berlec, 2012). Analogous techniques in metaproteomics were developed to study all proteins expressed by microbial communities and recovered directly from complex environmental samples (for a review, see Siggins *et al*., 2012). These studies allow functional genes and metabolic pathways to be tracked as well as being able to identify specific proteins. Knief *et al*. (2011) reviewed applications of metaproteomics to plant-associated bacterial communities using techniques of high-throughput identification of proteins by tandem mass spectrometry. A study with combined metagenomic and metaproteomic approaches (community proteogenomics) to analyse the physiology of bacterial phyllosphere communities *in situ* was initially carried out by Delmotte *et al*. (2009).

Berlec (2012) addressed the commensal bacteria of the whole plant-associated microbiota in a recent review. The author critically examined the application of novel ‘–omic’ techniques and drew remarkable parallels between certain plant and human microbiome studies. He deduced directions for future research to regulate the entire plant-associated microbial community to produce probiotic benefits to plants. Undoubtedly, therefore, an improvement in our current knowledge of microbial phyllosphere ecology is necessary. Do innovative microbiological techniques provide the preconditions for new insights into plant microbiology? What do we gain by extending our investigations to all members of the phyllosphere microbiome, beyond the spectrum of cultivable microorganisms? The objectives of this review are (1) to query the term ‘nonculturable’ and to estimate the relationship between culturable and the total number of microorganisms in the phyllosphere, and (2) to compile results of studies in phyllosphere microbiology based on culture-independent methods over the last 4 years. Particular attention is given to the developments in (i) fundamental questions regarding biodiversity and variability in microbial phyllosphere communities, (ii) linking the microbial diversity with actual functions of individual cells, populations and communities, (iii) the knowledge of interactions between plants and microorganisms and those between various microorganisms, and (iv) the biology of enteric human pathogenic bacteria colonizing leafy vegetables.

## Culturable and ‘nonculturable’ microorganisms in the phyllosphere

Rastogi *et al*. (2010) determined the total bacterial abundances in the same samples of leaves of field-grown lettuce by real-time quantitative PCR (qPCR) and by counting the number of colony-forming units (CFUs) on agar plates. The authors found that only 0.1–8.4% of the total bacterial population were cultivable. Unfortunately, studies such as these, which compare the densities of phyllospheric bacteria determined by culture-dependent and culture-independent methods, have been scarce until now (Table 1). However, the few results available to us indicate that the portion of cultivable bacteria in the phyllosphere is often, but not always, in the range of those in other environments too – typically 0.1–5% (Ritz, 2007).

**Table 1:** Direct comparison of culture-dependent (plate counts) and culture-independent estimations of the abundance of bacteria in the phyllosphere

Culture-independent methods	Objects	Portions of culturable bacteria*	Reference
qPCR	Spinach (*Spinacia oleracea*) Watercress (*Circhorium endivia*)	0.5% 0.8%	Ruppel *et al*. (2008), unpublished cell numbers
qPCR	Field-grown lettuce (*Lactuca sativa*)	0.1–8.4% 1–6%	Rastogi *et al*. (2010) Rastogi *et al*. (2012)
qPCR of *Salmonella enterica* serotype Typhimurium	Parsley (*Petroselinum crispum*)	2%	Kisluk & Yaron (2012)
qPCR	Seeds of spinach (*Spinacia oleracea*)	1%	Ponder *et al*. (2012)
Microscopic enumeration of DAPI-stained cells	Apple tree leaves	0.1–1%	Yashiro *et al*. (2011)
Microscopic enumeration of DAPI-stained cells	Leaves of *Arabidopsis thaliana*	3%	Reisberg *et al*. (2012), unpublished CFU, pers. commun.
Microscopic enumeration with Thoma chamber	Trifoliates of white clover (*Trifolium repens*)	10%	Stiefel *et al*. (2013)
Microscopy of fluorescent nuclear-stained cells			
Enumeration of total bacteria	Leaf sheaths of rice (*Oryza sativa*)	23–35%	Niwa *et al*. (2011)
Enumeration of living bacteria		30–50%	

* Plate counts (CFU) related to number of cells determined culture independently.

The detected high differences in microbial cell numbers between culturable and total number of organisms may partly be caused by the methods used. In particular, the qPCR technique is subject to the following weaknesses, which can possibly affect the reliability of its data:

1. False-positive signals from plant chloroplasts and mitochondria may be amplified with ‘universal’ bacterial primers (Rastogi *et al*., 2010).
2. DNA of dead cells in environmental samples is amplified by PCR (Degefu *et al*., 2009).
3. Differences in 16S rRNA gene copy numbers per cell may artificially lead to overrepresentation of some species (Rastogi *et al*., 2012).

The discrepancy between qPCR- and CFU-based determinations of bacterial population sizes in the phyllosphere is usually stronger than that based on microscopic enumeration vs. plate counting (Table 1). This is possibly an indication of the weaknesses of qPCR mentioned above. But, amplification of dead cells can explain this discrepancy just as little as incorrect calculations with different gene copy numbers per bacterial cell. The difference between microscopically enumerated bacterial cell numbers and CFU also persisted when exclusively living bacterial cells were microscopically counted (Niwa *et al*., 2011). The culturable portion of the total bacterial population was the highest with up to 50% in this case (Table 1). Another extreme case of differences in estimating the cultivable portion of the phyllospheric bacterial population has most recently been described by Stiefel *et al*. (2013): from a total of more than 100 transfers of individual bacterial cells from leaf washes into separate compartments, about two-thirds of the isolates grew in a liquid medium, whereas plating of dilutions from the same leaf washes on a solid medium resulted in a portion of cultivable bacteria of only 10% related to total microscopically counted cells (Table 1). Currently, based on the data in Table 1, it is hardly possible to give a general estimation of the portion of culturable bacteria in the phyllosphere.

An explanation for the ‘uncultivability’ of bacteria could be that they enter a ‘viable but nonculturable’ (VBNC) state. This is a reversible dormancy phase in bacteria when they are unable to undergo a sustained cellular division on or in standard laboratory media (Oliver, 2010). This state can also be induced by stress-provoking environmental conditions such as the hostile ones on aerial plant surfaces (nutrient limitation, desiccation, variations in temperature and UV radiation). Bacteria entering the VBNC state maintain a low level of metabolic activity and can remain this way for long periods (McDougald *et al*., 1998).

The phenomenon of the VBNC state does indeed occur in the phyllosphere (for a review, see Wilson & Lindow, 2000). A study by Dinu & Bach (2011) showed that bacterial populations evolved towards VBNC may have implications regarding food safety; although they lost their cultivability under low temperatures, the highly infective *Escherichia coli* O157:H7 on lettuce leaves still produced verotoxins and thus retained their virulent potential.

On the other hand, more than half of the known bacterial phyla in the environment contain unculturable representatives (Schloss & Handelsman, 2005). This largely appears to be a question of available media and culture conditions (Nichols, 2007). The current culturing technologies do not adequately reproduce the natural environment in which the microorganisms normally grow (Ritz, 2007). So far, we do not know the suitable nutrient media and corresponding culture conditions for most microorganisms from environmental samples. Therefore, it seems that ‘nonculturable’ environmental microorganisms are better defined as being ‘not yet cultured’.

Yashiro *et al*. (2011) performed a unique study to quantitatively as well as qualitatively assess the bacterial community on apple leaves by culture-based and culture-independent methods with the same samples. Serially diluted leaf extract was plated onto a growth agar to determine the CFUs of culturable bacteria, whereas the total number of these microorganisms in the same extract was enumerated microscopically (Table 1). The cell counts determined microscopically were at least 100–1000 times greater than were estimated by culturing. Sequences of the 16S rRNA gene from each of the 309 isolates from agar plates and 317 clones derived from DNA of cells in the leaf extracts were compared with GenBank sequences. The phylogenetic trees of the bacterial communities assessed by culture-based vs. culture-independent methods revealed differences. The richness of operational taxonomic units derived from clones was greater than found in those of isolates, despite the relatively modest number of sequences examined. The orders *Bacteroidales*, *Sphingobacteriales*, *Myxococcales* and *Enterobacteriales* represented in clone libraries were absent among cultured isolates. There was an overlap of operational taxonomic units between isolates and clones. The isolates, however, were not merely a subset of bacteria represented in clone libraries, because the order *Actinomycetales*, which was prevalent among isolates, was absent in clone libraries. These differences were discussed as being results of the limitations and biases of the methods used.

A much deeper sampling depth than in the above-mentioned study is achieved in high-throughput pyrosequencing analyses of 16S rRNA gene amplicons. This commonly leads to the detection of many more and in the phyllosphere also less frequently occurring taxa. Furthermore, species that had never been detected in culture-based phyllosphere studies or those that had been hitherto unknown are discovered. Investigations by Lopez-Velasco *et al*. (2011) using pyrosequencing of 16S rRNA gene amplicons set an example: of *c*. 8800 unique sequences examined from fresh spinach leaves, 75% were not present in existing databases. Another example was the study by Takahashi *et al*. (2011): when they analysed the endophytic bacterial community in rice by pyrosequencing 16S rDNA gene amplicons from intercellular fluid, the authors found that 63% of 2331 reads did not match annotated species in the nucleotide database. However, *c*. 70% of 158 980 bacterial V6 sequences derived from *Tamarix* tree leaves from various regions of the world that were analysed by tag pyrosequencing could be assigned to 788 genera (Finkel *et al*., 2011).

The phenomenon of ‘uncultivability’ seems not to be restricted to bacteria. Based on pyrosequence data from rITS1 amplicons derived from beech leaves, Cordier *et al*. (2012) concluded that many of the ascomycete taxonomic units in their data set represent uncultivable fungi too.

At present, we do not know how to culture these ‘not yet culturable’ microorganisms nor do we understand their significance and functionality in ecosystem processes. The available cultural techniques only assess parts of the total microbial populations. Relating just this fraction to any functions in environmental processes is unlikely to be informative (Ritz, 2007). This limits our understanding of microbial ecology in the phyllosphere. Nichols (2007) suggests solving this problem by an improved cultivation-based microbial ecology because this may provide information about communities that cannot be obtained from sequencing alone. She argues that community metagenomic efforts suffer from difficulties in assembling the genomes of community members and assigning the genes to individual organisms. However, microbial cultivation provides direct access to genomes of environmental isolates and means that the theoretical findings of metagenomics can be examined. Overall, the author appeals for improvements in both cultivation-dependent and cultivation-independent techniques to assess microbial communities synergistically.

## Studies of microbial phyllosphere ecology based on culture-independent approaches

### The biodiversity and variability of microbial phyllosphere communities

Culture-independent methods, especially high-throughput sequencing techniques, have the advantage of being able to almost comprehensively assess the genetic diversity of complex microbial communities in their natural environments. Consequently, studies using molecular methods such as 16S rRNA gene amplicon sequencing (Table 2) and whole metagenome shotgun sequencing (Delmotte *et al*., 2009; Knief *et al*., 2012) for analysing the microbial communities on and in above-ground plant parts have made a special contribution to extending our knowledge of the microbial diversity in the phyllosphere over the last years. Most techniques used in phyllosphere biodiversity studies have been based on 16S rRNA gene amplicon pyrosequencing (Table 2). In spite of the known bias inherent in PCR methods, none of the presently available primers is able to amplify all sequences from the corresponding domain (Bodenhausen *et al*., 2013); these studies have added enormously to the list of known microbial taxa occurring in the phyllosphere. For example, Finkel *et al*. (2011) detected 788 bacterial and 64 eukaryotic genera on leaves of the salt-secreting desert tree, *Tamarix,* from various geographic locations. A total of 478 genera could be identified from studying the changes in the bacterial community on field-grown lettuce along geographical and seasonal scales (Rastogi *et al*., 2012). Jumpponen & Jones (2010) determined 165 genera in a study of the seasonal dynamics of the fungal community on leaves of *Quercus macrocarpa*. In comparison, 37 named bacterial and 12 fungal genera were found in a comprehensive culture-based taxonomic study in the phyllosphere of spring wheat (Legard *et al*., 1994). This seems to be another example where depth of sampling may be much less in the culture-based studies (see in chapter Culturable and ‘nonculturable’ microorganisms in the phyllosphere).

**Table 2:** Studies on the microbial diversity in the phyllosphere using culture-independent approaches

Aims of investigations	Objects	Methods	Results	References
Bacterial and fungal epi- and endophytic phyllosphere communities affected by long-term summer drought	Leaves of *Quercus ilex*	T-RFLP analysis	Richness and diversity decreased on the surface and in the interior of leaves in summer. Drought promoted TRF richness, especially that of epiphytic microorganisms	Peňuelas *et al*. (2012)
Endophytic bacterial communities influenced by plant species, season and location	Leaves of common plant species of a tallgrass prairie	T-RFLP analysis	Distribution of endophytic bacteria was mostly related to host species followed by sampling dates and location.	Ding *et al*. (2013)
Annual and seasonal variations in bacterial community structure	Leaves of a single tree of *Magnolia grandiflora*	DGGE and sequencing of 16S rRNA gene clone libraries	Distinct seasonal patterns of bacterial communities were not predictable from year to year.	Jackson & Denney (2011)
Diversity of phyllopheric bacteria and its relationship with airborne bacteria	Leaves of several Mediterranial perennial and herbaceous evergreen plants	DGGE and sequencing of 16S rRNA gene clone libraries	Bacteria on leaves were not related to bacteria in the air. Plant species had strong influence on the composi tion of bacterial community	Vokou *et al*. (2012)
Quantification of the spatial variability in fungal assemblages	European beech (*Fagus sylvatica*) in different spatial scales (tree, branch, group of leaves, individual leaf)	Capillary electrophoresis–SSCP and pyrosequencing of ITS amplicons	Variability was highest between indi vidual leaves. Dissimilarity between fungal assemblages correlated rather with genetic distance than with geographic distance between trees.	Cordier *et al*. (2012)
Composition of bacterial populations influenced by tree species and geographic locations	Leaves of 56 tree species, needles of *Pinus ponderosa* in various locations of the world	Pyrosequencing of 16S rRNA gene amplicons	Bacterial diversity was greater across than within species. Little influence of geographic differences across continents. Patterns of bacterial communities' structures were predictable from the relateness of the trees.	Redford *et al*. (2010)
Composition of bacterial populations across an ele vational gradient	Leaves of *Weinmannia* trees in the eastern Andes of Peru	Pyrosequencing of 16S rRNA gene amplicons	Unlike plants and animals, bacteria did not exhibit an elevational gradient in their diversity.	Fierer *et al*. (2011)
Composition and diversity of bacterial communities	Leaves of six tropical tree species common in rainforests of the Malay Peninsula	Pyrosequencing of 16S rRNA gene amplicons	Tropical trees had a distinctive bacte rial phyllosphere community, which was not greatly different from that of temperate or subtropical trees.	Kim *et al*. (2012)
Comparison of epiphytic and endophytic bacterial communities	Leaves of *Arabidopsis thaliana*	Pyrosequencing of 16S rRNA gene amplicons	Richness was lower in epiphytic than in endophytic samples. Gamma- proteobacteria (*Pseudomonas*) were dominant in the epiphytic community.	Bodenhausen *et al*. (2013)
Composition of the bacterial phyllosphere community depending on geographical distances	Leaves of *Tamarix aphylla* trees along a 500 km tran sect with uniform environmental conditions across the Soronan desert	Pyrosequencing targe ting V4–V6 regions of 16S rRNA genes	Community similarity declinedsigni ficantly with geographic distance, the most important parameter that affects the community composition under similar environmental conditions.	Finkel *et al*. (2012)
Composition of the microbial phyllosphere community depending on the geographic location	Leaves of different *Tamarix* tree species in Medite rennian and Dead Sea hypervariable regions and two locations in the USA	Pyrosequencing of 16S/18S rRNA gene amplicons	Microbial communities on different *Tamarix* species were highly similar in the same location, whereas trees of the same species growing in different climatic regions hosted dis tinct microbial communities.	Finkel *et al*. (2011)
Seasonal dynamics of the composition of fungal populations	Leaves of *Quercus macrocarpa* in urban and nonurban environ ments	Pyrosequencing of ITS2 amplicons	Fungal communities were lower in diversity and richness on urban trees. Seasonal patterns of fungal communities were predictable.	Jumpponen & Jones (2010)
Spatiotemporal variations in bacterial community composition	Field-grown Romaine lettuce	qPCR of total bacterial population, pyrosequencing of 16S rRNA gene amplicons	Variability in bacterial communities' composition on plant leaves was determined by season, field location and environmental conditions.	Rastogi *et al*. (2012)
Analysis of the bacterial community and comparison with those of previously analysed plant species	Aerial parts of rice (*Oryza sativa*)	DGGE and sequencing of 16S rRNA gene clone libraries, whole metagenome shot gun sequencing	Dominance of Alphaproteobacteria (*Rhizobium* and *Methylobacterium*) and Actinobacteria (*Microbacterium*). The complexity of this community was comparable with those of other plants.	Knief *et al*. (2012)

Species richness in fungi is one order of magnitude lower than that of bacteria (Finkel *et al*., 2011). However, species of the predominating genera *Alternaria*, *Phoma* and *Aureobasidium* detected using ITS2 amplicon pyrosequencing on oak leaves by Jumpponen & Jones (2010) had also been isolated as the most common species on sugar beet and wheat in earlier plate-culture studies (Thompson *et al*., 1993; Legard *et al*., 1994).

It seems that our view of the microbial diversity in the phyllosphere did not become entirely new since culture-independent methods were introduced, but our knowledge of species richness has been considerably extended towards less frequently occurring microbial taxa.

Studies of microbial diversity by 16S rRNA gene amplicon sequencing have also revealed plant species-specific, geographic and seasonal influences on the variability of the microbial communities' composition (Table 2). Hunter *et al*. (2010), Redford *et al*. (2010), Vokou *et al*. (2012) as well as Ding *et al*. (2013) have recognised the species of the plant as a main determinant of the composition of phyllosphere communities.

Recent studies by Rastogi *et al*. (2012), Qvit-Raz *et al*. (2012) and Finkel *et al*. (2011, 2012) have investigated the significance of geographical locations and their specific environmental conditions as decisive factors driving the variability in microbial phyllosphere communities. Finkel *et al*. (2011) concluded from their study on bacteria and fungi colonizing the leaves of *Tamarix* trees in desert regions that the climatic conditions in different geographic regions are more important factors driving variability than the host species. However, in a robust biogeographical analysis of bacterial communities colonizing the leaves of *Tamarix aphylla* trees along a 500-km transect in a desert with relatively uniform climatic conditions in the south-western United States, Finkel *et al*. (2012) revealed a strong distance–decay relationship caused by dispersal limitations. The authors concluded that the geographical distance is the most important parameter affecting the composition of the bacterial community in the absence of environmental differences. Qvit-Raz *et al*. (2012) analysed denaturing gradient gel electrophoresis (DGGE) patterns of the bacterial 16S rRNA genes amplified from leaves of the same tree species situated at various sites in the Dead Sea region. It was observed that similarities between the bacterial communities diminished when the distance between the sampling sites was increased, thus confirming in this study too that the geographical location plays a major role in determining the population composition.

Leaves of trees in both temperate and tropical zones have their distinctive bacterial phyllosphere communities (Redford *et al*., 2010; Kim *et al*., 2012). However, tropical tree communities do not greatly differ from their temperate or subtropical counterparts and have relatively abundant lineages within the Alpha- and Gammaproteobacteria, Actinobacteria and Bacteroidetes. However, beyond these phyla, it is characteristic for Acidobacteria to dominate in tropical phyllosphere communities, whereas these are absent or at a low abundance on trees in other regions (Kim *et al*., 2012).

Bacteria as well as fungi are subject to seasonal dynamics (Jumpponen & Jones, 2010; Jackson & Denney, 2011; Rastogi *et al*., 2012; see Table 2). Peňuelas *et al*. (2012) assessed T-RFLP profiles from bacteria and fungi on the surface and in the interior of leaves of *Q. ilex* in Mediterranean forests from the wet spring to the dry summer season. They confirmed a strong seasonal influence on the richness and diversity of the microbial phyllosphere community.

The new insights into microbial diversity in the phyllosphere have not yet answered all the remaining questions and, furthermore, have raised new ones: firstly, it is largely unknown whether differences in population composition have consequences for different metabolic functionalities (Hunter *et al*., 2010; Peňuelas *et al*., 2012). Additionally, the identification of the important drivers of microbial community structure has not yet been completed (Vorholt, 2012). Is it the location *per se* or does the local climate contributes to the observed variations in community composition (Rastogi *et al*., 2012)? Comparing the microbial succession on plants under controlled environmental conditions with those being grown under field conditions, as Redford & Fierer (2009) have already suggested, could be the key to resolving this question.

### Functional structures and metabolic diversity in microbial phyllosphere communities

Studies using cultivation-independent techniques to identify actual functions of individual cells, populations or whole communities are scarce in plant microbiology (Berlec, 2012). However, outstanding examples are the metaproteogenomic approaches to investigate the physiology of bacteria colonizing leaves of soya bean, clover and *Arabidopsis thaliana* by Delmotte *et al*. (2009) and that of bacteria and archaea living in the rhizosphere and phyllosphere of rice by Knief *et al*. (2012). Shotgun sequencing and liquid chromatography high-accuracy mass spectrometry were performed in the latter study to collect metagenomic and metaproteomic information, respectively. A total of 4308 different proteins were identified in the phyllosphere, of which 62% were of bacterial and archaeal origin. A number of microbial proteins were recognized to be specific for life in the phyllosphere (Table 3). Although a large diversity of *nif*-genes was also found in the phyllosphere, the protein dinitrogen reductase was exclusively detected in the rhizosphere. And furthermore, enzymes involved in the methanol-based methylotrophy were detectable in both rhizosphere and phyllosphere but were prevailing in the phyllosphere, where the one-carbon metabolism is associated with the genus *Methylobacterium*, a dominant member of the bacterial phyllosphere community (Ikeda *et al*., 2010a). Rice plants release methane formed by archeae in the rhizosphere via aerial plant parts. However, enzymes with methane-oxidizing activity could not be detected in the phyllosphere metaproteome, and there was no evidence of the encoding genes in the corresponding metagenome. Hence, it seems that methanotrophic Alpha- and Gammaproteobacteria that were detected in phyllosphere samples of this study play no role in methane utilization. The findings of this study impressively demonstrate the advantage of functional metaproteogenomic approaches over metagenomic techniques alone for inferring the *in situ* physiology of microbial communities.

**Table 3:** Functional structure and metabolic diversity in phyllospheric microbial communities detected by culture-independent approaches

Aims of investigations	Objects	Methods	Results	References
Identification of microbial genes encoding bioactivity in endophytes	Leaves and stems of 30 traditional Chinese medical herbs	PCR using primers targeting genes of polyketide synthases (PKS) and nonri bosomal peptide syntheta ses (NRPS)	Presence of PKS and/or NRPS genes in 36% of the samples indicated possible bioactivity by endophytes in the herbs.	Miller *et al*. (2012)
Impact of biofilm formation by *E. coli* O157:H7 on metabolic activity of the phyl lospheric community	Leaf lysate of spinach (*Spinacia oleracea*)	GeoChip microarray	Abundance of genes involved in C-, N- and P-cycling decreased in result of the biofilm formation.	Carter *et al*. (2012)
Detection of diverse microbial rhodopsins	Leaves of *Tamarix nilotica, Glycine max*, *Arabidopsis thaliana,Trifolium repens*, *Oryza sativa*	Whole metagenome shotgun sequencing	Sequences of *rplA*, *rplC*, *rplD*, *rpoA, rpoB* and *rspJ* genes in dicated the existence of micro bial rhodopsins in all phyllospheric metagenomes.	Atamna-Ismaeel *et al*. (2012a)
Detection of anoxygenic phototrophic bacteria	Leaves of *Tamarix nilotica*, *Arabidopsis thaliana, Trifolium repens*, *Pinus silvestris*,*Poa pratensis*	Whole metagenome shotgun sequencing	*pufM*, *pufL* and *bchY* genes of phototrophs were detected in all phyllospheric metagenomes except for that of *Tamarix*. Anoxygenic phototrophic community included methylobacteria and diverse Proteobacteria.	Atamna-Ismaeel *et al*. (2012b)
Identifying the major physiological traits of dominant microorganisms	Aerial parts of rice (*Oryza sativa*)	Whole metagenome shotgun sequencing, protein analyzation by HPLC-ESI-MS/MS	Protein families specific for phyllosphere communities involved in substrate uptake, response to reactive oxygen species, fasciclin, methanol utilization and invasion-associated locus B were detected.	Knief *et al*. (2012)

Pyrosequencing of metagenomes derived from the surfaces of various terrestrial plants recently revealed phototrophy in phyllosphere bacteria (Table 3). Microbial rhodopsin sequences (Atamna-Ismaeel *et al*., 2012a) and a diverse community of anoxygenic phototrophic bacteria (Atamna-Ismaeel *et al*., 2012b) were detected in different phyllospheric metagenomes. The ability to use light as an additional energy source might be advantageous for epiphytic bacteria at times of nutrient deficit (Vorholt, 2012).

However, it should be noted that investigations into the functions of phyllospheric microbial communities based on culture-independent methodologies are currently the exception rather than the rule. Although functional studies are carried out using numerous molecular biology methodologies and genetic techniques, their usual starting point is based on the cultures of individual microorganisms that have already been isolated from plant materials. In spite of ongoing advances in genome assembly algorithms, the cultivation of unique isolates has the distinct advantage of sequencing in less time and with fewer resources than metagenomes and assigning the genes is also easier (Nichols, 2007).

Overall, the exploration of metabolic diversity and functions in microbial phyllospheric communities remains a very complex area and can be performed using both cultivation-based and culture-independent approaches. The question of whether changes in the composition of communities are reflected in the actual functions of these communities or their individual members is still yet to be answered in many respects.

### Interactions between plants and microorganisms in the phyllosphere

The plant's genotype appears to be an important factor in determining the structure of microbial communities in the phyllosphere (Whipps *et al*., 2008). However, the phenotypic characteristics or possible mechanisms expressed in the plant to shape the populations of their microbial epiphytic and endophytic colonizers are so far largely unknown. Clarifying the interactions between plant genotypes and the phyllosphere microbial community as well as identifying the plant genes that are crucial to these microorganisms are prerequisites for managing the phyllosphere microbiota, for example, in terms of disease resistance and plant health.

The relationship between plant characteristics at cultivar level (and their underlying genotypes) and developing phyllosphere microbial populations has been addressed in studies by Hunter *et al*. (2010) and Balint-Kurti *et al*. (2010) (Table 4a). Hunter *et al*. (2010) investigated the influences of physical, chemical and physiological plant properties on the bacterial population profiles for 26 lettuce accessions representing a wide range of genetic diversity. Their phyllospheric bacterial community structures were analysed through T-RFLP profiling. Parameters of leaf morphology (shape, margin crenulations, surface blistering, distribution of veins and hydathodes, and the density of epidermal cell wall junctions) as well as leaf chemistry (levels of leaf wax, nitrogen, potassium, calcium, magnesium, phenolic compounds, soluble carbohydrate and water content) were recorded to determine plant morphotypes. Multiresponse permutation procedure analyses indicated significant differences between T-RFLP profiles from different plant morphotypes. A more detailed breakdown of the structure of the bacterial populations was obtained by constructing 16S rRNA gene clone libraries from total DNA extracts from three representative lettuce accession lines. These clone libraries were found to be significantly different in their sequences and in the bacterial genera represented. Overall, leaf properties were shown to play important roles in differentiating bacterial populations in the phyllosphere. Factors that were significantly associated with the differences between bacterial populations were the levels of soluble carbohydrate, water content and leaf blistering.

**Table 4:** (a) Interactions between host plants and phyllospheric microorganisms studied using culture-independent approaches and (b) interactions between phyllospheric microorganisms investigated cultivation independently

Aims of investigations	Objects	Methods	Results	References
(a)				
Influence of plant genetic traits on the diversity of the epiphytic bacterial community and on plant disease susceptibility	Leaves of maize (*Zea mays*) recombinant inbred lines	T-RFLP analysis, QTL analysis of the maize chromosome	Loci (QTL) determining the leaf bacterial diversity were identified in the maize chromosome. A genetic correlation between bacterial diversity and disease susceptibility was detected.	Balint-Kurti *et al*. (2010)
Influence of leaf properties on within-species variation in bacterial population diversity and structure	26 lettuce (*Lactuca* spec.) accessions	T-RFLP and sequencing of 16S rRNA gene clone libraries	Levels of soluble carbohydrate, water content and leaf bliste ring significantly determined the composition of the bacterial population.	Hunter *et al*. (2010)
Effects of plant gene mutations on the composition of stem-associated bacterial community	Stems of soya beans (*Glycine max*) wild type and non-nodulated and hypernodulated mutants	Sequencing of 16S rRNA gene clone libraries	Genetic alteration in the nodulation/mycorrhization signalling pathways also altered the plant microbial communities, additionally to rhizobia and mycorrhizae.	Ikeda *et al*. (2010b)
Effects of transgenic changes in plants on the composition of bacterial and fungal communities	Leaves of transgenic *(Bacillus thuringiensis* protein) cotton at various growth stages	DGGE and sequencing of 16S/18S rRNA gene clone libraries	Due to transgenic *Bt* cotton, bacterial diversity was decreased at the budding stage only. Fungal diversity and abundancy slightly increased at different stages.	Pan *et al*. (2012)
Effects of leaf trichomes on the microbial community composition on leaves	Leaves of *Arabidopsis thaliana* wild type and a trichomeless mutant	Microscopic enumeration of DAPI-stained cells, DGGE and sequencing of 16S rRNA gene clone libraries	Cell counts in leaf washings did not significantly differ between the plant lines. The composition of cuticular waxes on leaves of mutant and wild type was nearly similar. Trichomes did not affect bacterial diversity.	Reisberg *et al*. (2012)
(b)				
Assessing the relative proportions of bacterial genera on various lettuce accessions	26 lettuce (*Lactuca* spec.) accessions	T-RFLP and sequencing of 16S rRNA gene clone libraries	Bacteria of the genus *Enterobacter* affected the leaf colonization by those of the genus *Erwinia*.	Hunter *et al*. (2010)
Effects of black fungi on the bacterial community under conventional and organic viticultural conditions	Leaves, shoots and grapes of grapevine (*Vitis vinifera*)	qPCR of *Aureobasidium pullulans*, SSCP and sequencing of 16S rDNA gene clone libraries	No differences in bacterial diversity under organic viticulture with black fungi enrichment and on conventionally managed plants.	Grube *et al*. (2011)
Influence of acyl-homoserine lactones (AHLs) on the composition of the bacterial community	Leaves of tobacco (*Nicotiana tobacum*)	DGGE and sequencing of 16S rRNA gene clone libraries, phosphate lipid fatty acid analyses	AHLs induced variability in the composition of the bacterial community. In particular, Grampositive species, which do not use these compounds for QS, were affected.	Lv *et al*. (2012)

Balint-Kurti *et al*. (2010) revealed differences in the diversity of the epiphytic bacterial populations on recombinant inbred lines of maize and assessed the plant determinants of the microbial communities by comparing the genetic architecture of traits in the different plant genotypes. The authors identified six chromosomal regions (QTL) controlling the epiphytic bacterial diversity. These loci had a significant overlap with those controlling southern leaf blight (SLB) fungal disease resistance in maize. The maize genome sequences in the QTL regions contained a gene encoding the glutamate decarboxylase, an enzyme in the biosynthesis of the gamma-aminobutyric acid mediating plant interactions with other organisms. The authors found a genetic correlation between low phyllosphere diversity and SLB disease resistance. This correlation might be caused by underlying plant traits that possibly encourage the establishment of beneficial bacterial species. It has been suggested that structural and metabolic differences in leaves caused selective microbial growth in this study too. Concurrently, Balint-Kurti *et al*. (2010) and also Hunter *et al*. (2010) concluded from their studies that leaf structure and chemistry should be breeding targets for managing the phyllosphere bacterial population and reducing the growth of pathogens on vegetables and crops. Furthermore, it is conceivable that variability in the spatial distribution and diversity of epiphytic microorganisms could be driven by the presence or absence of microniches on the leaf. In particular, the bases of trichomes, stomata, epidermal cell wall junctions and grooves along veins are considered to be hot spots for bacterial leaf colonization (Beattie & Lindow, 1999). However, cell counts and DGGE patterns in leaf washings from the A*rabidopsis thaliana* trichome-occupied ecotype Col-0 and its trichomeless *gl1* mutant showed only minor differences, suggesting that trichomes *per se* do not affect the bacterial community in the *Arabidopsis* phyllosphere (Reisberg *et al*., 2012).

Pan *et al*. (2012) examined the relationship between phyllospheric bacteria and fungi and the expression of the *Bacillus thuringiensis* (*Bt*) Cry1Ac protein in transgenic cotton. DGGE profile data and sequences of 16 and 18S rRNA gene fragments suggested that fungi may be more susceptible to Cry1Ac protein than bacteria (Table 4a). However, it seems that changes in environmental conditions and different growth stages contribute more to variations in microbial communities than the expression of the *Bt* toxin.

The short list of examples of studies on plant–microorganism interactions that are aiming to decipher the control of microbial phyllosphere communities by the host plants themselves (see Table 4a) indicates that research activity in this field is merely moderate at present. This appears rather surprising because there is an urgent need to improve our knowledge of these interactions to manipulate biotechnologically the phyllosphere community. The methodological preconditions are given for both microbiology and plant science, as shown in the study by Balint-Kurti *et al*. (2010).

Strikingly, the interactions are examined unilaterally from plant to microorganisms in all the studies listed in Table 4a. However, in connection with the expression of numerous phenolic compounds associated with plant defence against pathogenic bacteria and fungi, Hunter *et al*. (2010) asked the question, whether variation in the phyllosphere population has also any reciprocal effect on the host plant. Surely, it would also be appropriate to examine the effects of phyllosphere microorganisms on plants in addition to phytopathogenic activity. In this way, new approaches in biofertilization and phytostimulation could possibly be discovered.

### Interactions between microorganisms in the phyllosphere

Knowledge of the mechanisms, genes and compounds involved in interactions between microorganisms of the plant microbiome is essential for practical use in biological plant protection. Biological control agents are inherently cultivable microorganisms, and their cultivation is a prerequisite to apply biocontrol strains. Therefore, and for reasons already described in the section ‘Functional structures and metabolic diversity in microbial phyllosphere communities’ above, studies on microbial agents are usually based on cultivation in suitable media. However, a few other studies, which aimed at a more comprehensive assessment of the microbial phyllosphere communities influenced by microorganism–microorganism interactions, were also based on culture-independent approaches (Table 4b). Lv *et al*. (2012) identified several *N*-acyl-homoserine lactones (AHLs) used in Gram-negative Proteobacteria as quorum sensing (QS) molecules to regulate density-dependent mechanisms in bacterial communities in the tobacco phyllosphere and determined changes in the composition of this community by DGGE and phospholipid fatty acid analyses. It was suggested that pseudomonads and other AHL-producing Gammaproteobacteria utilize QS-dependent mechanisms to ensure their survival over other epiphytic residents in the nutrient-poor phyllosphere. Hence, AHL QS signals occurring naturally in the phyllosphere could play a role in the interactions between plant-associated bacteria. It is possible that AHL QS signals could be used to suppress pathogens in the phyllosphere of crops.

Detailed analyses of functional genes in a microbial community of a biofilm established in spinach leaf lysate, which was impaired by co-inoculation with *E. coli* O157:H7, supported the hypothesis that competition for nutrients is the primary mechanism of interactions between phyllospheric microorganisms (Carter *et al*., 2012). Data recorded by GeoChip probes indicated that *E. coli* O157:H7 competed for carbon mainly with Actinobacteria, Proteobacteria, Basidiomycota and uncultured fungi and for nitrogen with Proteobacteria, Actinobacteria and uncultured bacteria.

When studying the composition of bacterial communities colonizing the leaves of preharvest field-grown lettuce by pyrosequencing of 16S rRNA gene amplicons, Rastogi *et al*. (2012) found correlations between the abundance of *Xanthomonas campestris* pv. *vitians*, the causative agent of the bacterial leaf spot, and the presence or absence of other phyllosphere bacteria. It is possible that strains of the genus *Alkanindiges* act as facilitators, and those of *Bacillus*, *Erwinia* and *Pantoea* operate as antagonists of the pathogen.

The interaction between microorganisms in the phyllosphere is surely one of the most important issues raising questions that have to be answered before practical applications can be established. However, the research is far from complete. At present, we do not know whether the abundance of plant pathogens is a function of interactions between phyllosphere microorganisms or of the plant genotype. This needs to be clarified through further in-depth studies.

### The colonization and persistence of potential human pathogenic bacteria in the phyllosphere of leafy greens

In their large culture-independent survey of leaf surface microbiology, Rastogi *et al*. (2012) found an overrepresentation of *Enterobacteriaceae* including many culturable coliforms in the summer samples of field-grown lettuce, reflecting that this is a natural part of the lettuce microbiota instead of being accounted for by faecal contamination.

Enterohemorrhagic *E. coli* and nontyphoidal *Salmonella* are enteric human pathogens and do not naturally occur in plants. However, they have been associated with multiple outbreaks of foodborne illness caused by the consumption of fresh-cut leafy vegetables (Teplitski *et al*., 2011). Sources of these enteric foodborne pathogens are faeces and manure as well as contaminated water and soil. Current research is directed towards the ecology of foodborne pathogens and their routes of contamination as well as towards developing novel approaches for inhibiting or inactivating these bacteria (Critzer & Doyle, 2010).

Enterohemorrhagic *E. coli* and nontyphoidal *Salmonella* are intrinsically culturable, but they can enter the VBNC state (Dinu & Bach, 2011). Studies on their distribution and persistence are mainly based on cultivation methods. The fate of these species on inoculated leafy greens is usually observed with antibiotic-resistant derivative strains that can be detected on semi-selective indicator media. In this way, the contamination of vegetables by enteric bacteria via irrigation and their survival as well as interactions with plant tissues and the inherent phyllospheric microbiota has been demonstrated (e.g. Barak *et al*., 2011; Moyne *et al*., 2011; Lopez-Velasco *et al*., 2012; Quilliam *et al*., 2012). Alternatively, the distribution and persistence of these bacteria can also be observed microscopically using inoculants adapted to express genes for green fluorescent proteins (Golberg *et al*., 2011; Kroupitski *et al*., 2011.

Table 5 shows a few examples of studies on human pathogenic bacteria in the phyllosphere of vegetables based on culture-independent approaches. Studying the diversity of bacteria colonizing leafy greens with special reference to enteric bacteria by pyrosequencing of 16S rRNA gene amplicons has the advantage of providing a broader insight into the variations between the total communities (Lopez-Velasco *et al*., 2011; Telias *et al*., 2011). Such an analysis revealed a bacterial community consisting of 11 phyla on spinach leaves (Lopez-Velasco *et al*., 2011) compared with a previous study using sequence analysis of corresponding DGGE bands, which detected only six phyla under the same storage conditions (Lopez-Velasco *et al*., 2010). In their functional metagenomic study using microarrays, Carter *et al*. (2012, see also in Table 3) revealed the metabolic potential of the human pathogen *E. coli* O157:H7 in utilizing plant nutrients, which is significant to its persistence on plants.

**Table 5:** Enteric human pathogenic bacteria in the phyllosphere of leafy greens detected by culture-independent approaches

Aims of investigations	Objects	Methods	Results	Reference
Persistence and spread of *Salmonella enterica* sv. Weltevreden	Spinach (*Spinacia oleracea*) grown in a climate chamber	qRCR of the *Salmonella invA* gene	*S*. Weltevreden was capable of persisting in soil, root and shoot for prolonged periods.	Arthurson *et al*. (2011)
Survival and distribution of *E. coli* on leafy greens under preharvest through postharvest conditions	Asian baby leaf vegetables: Tatsoi (*Brassica rapa* cv. *rosularis*), Mizuna (*B. rapa* cv. *japonica*), Red Chard (*Beta vulg*. cv. *cicla*)	qPCR of the *E. coli* O157:H7 *rfbE* gene	*E*. *coli* rapidly declined after inoculation, but low populations survived production and postharvest operations.	Tomás-Callejas *et al*. (2011)
Persistence of *Salmonella enterica* serotype Typhimurium following spray irrigation with contaminated water	Parsley (*Petroselinum crispum*) grown in a greenhouse	qPCR of *Salmonella sirA* gene	*S. *Typhimurium persisted for 48 h on the leaves, if the irrigation water contained about 300 cells mL^−1^. It was detectable atleast for 4 weeks, if the water was contaminated by 8.5 log cells mL^−1^, but the population steadily declined.	Kisluk & Yaron (2012)
Effects of packaging and storage temperature on bac terial community and the fate of *E. coli* O157:H7	Freshly harvested and storaged spinach *(Spinacia oleracea*)	DGGE and sequencing of 16S rRNA gene clone libraries, qPCR of the *E. coli* virulence (*stxA*, *csgE*, *eaeA*) and stress response (*rpoS*, *sodB*) genes	Storage time and temperature affected the bacterial diversity and also virulence and stress response of *E. coli* O157:H7.	Lopez-Velasco *et al*. (2010)
Changes in bacterial diversity on leaves during storage at refrigeration temperatures	Freshly harvested and storaged spinach *(Spinacia oleracea*)	Pyrosequencing of 16S rRNA gene amplicons	Refrigerated conditions decreased the species richness, diversity and evenness. Growth inhibition of *Escherichia* spp.was achieved at 4 °C, but not at 10 °C storage.	Lopez-Velasco *et al*. (2011)
Effect of overhead irrigation with ground or surface water on the bacterial diversity	Fruit surface of field grown tomatoes (*Solanum lycopersicum*)	Pyrosequencing of 16S rRNA gene amplicons	Although, the two water sources had a significantly different bacterial composition, bacterial populations on the surface of fruits sprayed could not be dif ferentiated. *Pantoea* and *Enterobacter* were the most abundant genera on fruits.	Telias *et al*. (2011)

On the other hand, where quantitative evaluation of the persistence of a potential human pathogen following inoculation onto leafy vegetables is the aim of the study, qPCR targeting species-specific genes would be the recommended procedure (Arthurson *et al*., 2011; Kisluk & Yaron, 2012). Unlike plate counting, qPCR also includes cells at the VBNC stage, which can retain their virulent potential (Dinu & Bach, 2011).

Cultivation and culture-independent methods were combined in studies by Lopez-Velasco *et al*. (2010) and Tomás-Callejas *et al*. (2011) to investigate the effects of packaging and storage temperatures on the spinach epiphytic bacterial community and the fate of *E. coli* on diverse fresh-cut leafy greens under preharvest through to postharvest conditions, respectively. Lopez-Velasco *et al*. (2010) inoculated the fresh vegetable with a strain of *E. coli* O157:H7 transformed for GFP expression and kanamycin resistance. The total population of epiphytic bacteria was enumerated on culture plates. Changes in the bacterial community structure during storage were detected by sequencing DGGE bands of 16S rRNA gene amplicons. qPCR was applied for assessing the virulence and stress response genes of *E. coli* O157:H7 (Table 5). The study resulted in changes in the epiphytic microbiota with implications on the virulence and stress response of *E. coli* O157:H7 during storage. The fate of generic *E. coli* and *E. coli* O157:H7 during the production, harvest, processing and storage of leafy vegetables was reported in the study by Tomás-Callejas *et al*. (2011). Cocktails consisting of *E. coli* isolates and avirulent strains of *E. coli* O157:H7 were inoculated onto leafy greens in a greenhouse. Their survival was checked by genotyping the generic *E. coli* strains by REP-PCR and qPCR for the detection of *E. coli* O157:H7 (Table 5). Rapid declines in generic *E. coli* as well as *E. coli* O157:H7 were observed, but individual cells of both populations survived throughout the production and postharvest operations.

Fundamental questions regarding the ecology of enteric pathogens, their sources, persistence, distribution and routes of contamination of leafy greens have already been answered using culture and/or culture-independent methods. However, eliminating the risk of plant food contamination and subsequent human disease outbreaks requires clarifying the mechanisms by which enteric pathogens colonize plants and understanding how they can be inhibited or inactivated (Critzer & Doyle, 2010). The already existing set of diverse powerful methods should therefore be applied in further innovative research approaches.

## Concluding remarks

In general, the phyllosphere microbiology has benefited from culture-independent techniques, such as qPCR, microarray assays, 16S rRNA gene amplicon sequencing and whole metagenome shotgun analyses, over the last 4 years. Using metaproteogenomic approaches, Knief *et al*. (2012) demonstrated the great potential of these techniques for studying the physiology of phyllospheric microorganisms *in situ*. Leaf structure and chemistry have been shown to play important roles in differentiating bacterial populations in the phyllosphere and could be prospective breeding targets for managing the phyllosphere microbiota to reduce the growth of phyto- and human pathogens on vegetables and crops. However, the level of research activity into plant–microorganism interactions is merely moderate at present. More attention should be paid to the effects of phyllosphere microorganisms on plants in addition to those of phytopathogens and to the understanding of the crosstalk between microorganisms. Progress in phyllosphere microbiology is not necessarily connected with the application of culture-independent methods as shown in food microbiology to observe enteric human pathogens on fruit and vegetables. However, unlike plate counting, culture-independent techniques have the advantage of also assessing cells at the VBNC stage, which can retain their virulent potential. Consequently, improving and combining cultivation and culture-independent techniques present a challenge to our better understanding mechanisms of the microbial ecology in the phyllosphere.

## References

[b1] Ali N, Sorkhoh N, Salamah S, Eliyas M, Radwan S (2012). The potential of epiphytic hydrocarbon-utilizing bacteria on legume leaves for attenuation of atmospheric hydrocarbon pollutants. J Environ Manage.

[b2] Arthurson V, Sessitsch A, Jäderlund L (2011). Persistence and spread of *Salmonella enterica* serovar Weltevreden in soil and on spinach plants. FEMS Microbiol Lett.

[b3] Atamna-Ismaeel N, Finkel OM, Glaser F (2012a). Microbial rhodopsins on leaf surfaces of terrestrial plants. Environ Microbiol.

[b4] Atamna-Ismaeel N, Finkel O, Glaser F, Mering von C, Vorholt JA, Koblížek M, Belkin S, Béjà O (2012b). Bacterial anoxygenic photosynthesis on plant leaf surfaces. Environ Microbiol Rep.

[b5] Balint-Kurti P, Simmons SJ, Blum JE, Ballaré CL, Stapleton AE (2010). Maize leaf epiphytic bacteria diversity patterns are genetically correlated with resistance to fungal pathogen infection. Mol Plant Microbe Interact.

[b6] Barak JD, Kramer LC, Hao L-Y (2011). Colonization of tomato plants by *Salmonella enterica* is cultivar dependent, and type 1 trichomes are preferred colonization sites. Appl Environ Microbiol.

[b7] Beattie GA, Lindow SE (1999). Bacterial colonization of leaves: a spectrum of strategies. Phytopathology.

[b8] Berlec A (2012). Novel techniques and findings in the study of plant microbiota: search for plant probiotics. Plant Sci.

[b9] Bodenhausen N, Horton MW, Bergelson J (2013). Bacterial communities associated with the leaves and the roots of *Arabidopsis thaliana*. PLoS One.

[b10] Brandl MT, Cox CE, Teplitski M (2013). *Salmonella* interactions with plants and their associated microbiota. Phytopathology.

[b11] Carter MQ, Xue K, Brandl MT, Liu F, Wu L, Louie JW, Mandrell RE, Zhou J (2012). Functional metagenomics of *Escherichia coli* O157:H7 interactions with spinach indigenous microorganisms during biofilm formation. PLoS One.

[b12] Cordier T, Robin C, Capdevielle X, Desprez-Loustau M-L, Vacher C (2012). Spatial variability of phyllosphere fungal assemblages: genetic distance predominates over geographic distance in a European beech stand (*Fagus sylvatica*. Fungal Ecol.

[b13] Critzer FJ, Doyle MP (2010). Microbial ecology of foodborne pathogens associated with produce. Curr Opin Biotechnol.

[b14] Degefu Y, Virtanen E, Väyrynen T (2009). Pre-PCR processes in the molecular detection of blackleg and soft rot erwiniae in seed potatoes. J Phytopathol.

[b15] Delmotte N, Knief C, Chaffron S, Innerebner G, Roschitzki B, Schlapbach R, Mering von C, Vorholt JA (2009). Community proteogenomics reveals insights into the physiology of phyllosphere bacteria. P Natl Acad Sci USA.

[b16] Ding T, Palmer MW, Melcher U (2013). Community terminal restriction fragment length polymorphisms reveal insights into the diversity and dynamics of leaf endophytic bacteria. BMC Microbiol.

[b17] Dinu L-D, Bach S (2011). Induction of viable but nonculturable *Escherichia coli* O157:H7 in the phyllosphere of lettuce: a food safety risk factor. Appl Environ Microbiol.

[b18] Fierer N, McCain CM, Meir P, Zimmermann M, Rapp JM, Silman MR, Knight R (2011). Microbes do not follow the elevational diversity patterns of plants and animals. Ecology.

[b19] Finkel OM, Burch AY, Lindow SE, Post AF, Belkin S (2011). Geographical location determines the population structure in phyllosphere microbial communities of a salt-excreting desert tree. Appl Environ Microbiol.

[b20] Finkel OM, Burch AY, Elad T, Huse SM, Lindow SE, Post AF, Belkin S (2012). Distance-decay relationships partially determine diversity patterns of phyllosphere bacteria on *Tamrix* trees across the Sonoran desert. Appl Environ Microbiol.

[b21] Golberg D, Kroupitski Y, Belausov E, Pinto R, Sela S (2011). *Salmonella* Typhimurium internalization is variable in leafy vegetables and fresh herbs. Int J Food Microbiol.

[b22] Grube M, Schmid F, Berg G (2011). Black fungi and associated bacterial communities in the phyllosphere of grapevine. Fungal Biol.

[b23] Hunter PJ, Hand P, Pink D, Whipps JM, Bending GD (2010). Both leaf properties and microbe-microbe interactions influence within-species variation in bacterial population diversity and structure in the lettuce (*Lactuca* species) phyllosphere. Appl Environ Microbiol.

[b24] Ikeda S, Okubo T, Anda M (2010a). Community- and genome-based views of plant-associated bacteria: plant-bacterial interactions in soybean and rice. Plant Cell Physiol.

[b25] Ikeda S, Okubo T, Kaneko T, Inaba S, Maekawa T, Eda S, Sato S, Tabata S, Mitsui H, Minamisawa K (2010b). Community shifts of soybean stem-associated bacteria responding to different nodulation phenotypes and N levels. ISME J.

[b26] Innerebner G, Knief C, Vorholt JA (2011). Protection of *Arabidopsis thaliana* against leaf-pathogenic *Pseudomonas syringae* by *Sphingomonas* strains in a controlled model system. Appl Environ Microbiol.

[b27] Jackson CR, Denney WC (2011). Annual and seasonal variation in the phyllosphere bacterial community associated with leaves of the southern magnolia (*Magnolia grandiflora*. Microb Ecol.

[b28] Jumpponen A, Jones KL (2010). Seasonally dynamic fungal communities in the *Quercus macrocarpa* phyllosphere differ between urban and nonurban environments. New Phytol.

[b29] Kim M, Singh D, Lai-Hoe A, Go R, Rahim RA, Ainuddin AN, Chun J, Adams JM (2012). Distinctive phyllosphere bacterial communities in tropical trees. Microb Ecol.

[b30] Kisluk G, Yaron S (2012). Presence and persistence of *Salmonella enterica* serotype Typhimurium in the phyllosphere and rhizosphere of spray-irrigated parsley. Appl Environ Microbiol.

[b31] Knief C, Delmotte N, Vorholt JA (2011). Bacterial adaptation to life in association with plants – A proteomic perspective from culture to *in situ* conditions. Proteomics.

[b32] Knief C, Delmotte N, Chaffron S, Stark M, Innerebner G, Wassmann R, Mering C, Vorholt JA (2012). Metaproteogenomic analysis of microbial communities in the phyllosphere and rhizosphere of rice. ISME J.

[b33] Kroupitski Y, Pinto R, Belausov E, Sela S (2011). Distribution of *Salmonella typhimurium* in romaine lettuce leaves. Food Microbiol.

[b34] Legard DE, McQuilken MP, Whipps JM, Fenlon JS, Fermor TR, Thompson IP, Bailey MJ, Lynch JM (1994). Studies of seasonal changes in the microbial populations on the phyllosphere of spring wheat as a prelude to the release of a genetically modified microorganism. Agric Ecosyst Environ.

[b35] Lindow SE, Brandl MT (2003). Microbiology of the phyllosphere. Appl Environ Microbiol.

[b36] Lopez-Velasco G, Davis M, Boyer RR, Williams RC, Ponder MA (2010). Alterations of the phylloepiphytic bacterial community associated with interactions of *Escherichia coli* O157:H7 during storage of packaged spinach at refrigeration temperatures. Food Microbiol.

[b37] Lopez-Velasco G, Welbaum GE, Boyer RR, Mane SP, Ponder MA (2011). Changes in spinach phylloepiphytic bacteria communities following minimal processing and refrigerated storage described using pyrosequencing of 16S rRNA amplicons. J Appl Microbiol.

[b38] Lopez-Velasco G, Tydings HA, Boyer RR, Falkinham JO, Ponder MA (2012). Characterization of interactions between *Escherichia coli* O157:H7 with epiphytic bacteria *in vitro* and on spinach leaf surfaces. Int J Food Microbiol.

[b39] Lugtenberg BJJ, Chin-A-Woeng TFC, Bloemberg GV (2002). Microbe-plant interactions: principles and mechanisms. Antonie Van Leeuwenhoek.

[b40] Lv D, Ma A, Bai Z, Zhuang X, Zhuang G (2012). Response of leaf-associated bacterial communities to primary acyl-homoserine lactone in the tobacco phyllosphere. Res Microbiol.

[b41] McDougald D, Rice SA, Weichart D, Kjelleberg S (1998). Nonculturability: adaptation or debilitation?. FEMS Microbiol Ecol.

[b42] Miller KI, Qing C, Sze DMY, Neilan BA (2012). Investigation of the biosynthetic potential of endophytes in traditional Chinese anticancer herbs. PLoS One.

[b43] Moyne A-l, Sudarshana MR, Blessington T, Koike ST, Cahn MD, Harris LJ (2011). Fate of *Escherichia coli* O157:H7 in field-inoculated lettuce. Food Microbiol.

[b44] Newton AC, Gravouil C, Fountaine JM (2010). Managing the ecology of foliar pathogens: ecological tolerance in crops. Ann Appl Biol.

[b45] Nichols D (2007). Cultivation gives context to the microbial ecologist. FEMS Microbiol Ecol.

[b46] Niwa R, Yoshida S, Furuya N, Tsuchiya K, Tsushima S (2011). Method for simple and rapid enumeration of total epiphytic bacteria in the washing solution of rice plants. Can J Microbiol.

[b47] Oliver JD (2010). Recent findings on the viable but nonculturable state in pathogenic bacteria. FEMS Microbiol Rev.

[b48] Pan J, Cui M, Hu Q, Ma A, Bai Z, Yang D, Zhang H, Guo H, Qi H (2012). Multivariate analysis linkage of phyllospheric microbial community of transgenic cotton from SGK321 to Cry1Ac: a temporal expression dynamics. Afr J Microbiol Res.

[b49] Peňuelas J, Rico L, Ogaya R, Jump AS, Terradas J (2012). Summer season and long-term drought increase the richness of bacteria and fungi in the foliar phyllosphere of *Quercus ilex* in a mixed Mediterranean forest. Plant Biol (Stuttg).

[b50] Ponder M, Carder P, Lopez-Velasco G, Welbaum GE (2012). The development of spinach (*Spinacia oleracea*) phyllo-epiphytic bacterial community from seed through mature leaf stages is influenced by environment. Acta Hortic Issue.

[b51] Quilliam RS, Williams AP, Jones DL (2012). Lettuce cultivar mediates both phyllosphere and rhizosphere activity of *Escherichia coli* O157:H7. PLoS One.

[b52] Qvit-Raz N, Finkel OM, Al-Deeb TM, Malkawi HI, Hindiyeh MY, Jurkevitch E, Belkin S (2012). Biogeographical diversity of leaf-associated microbial communities from salt-secreting *Tamarix* trees of the Dead Sea region. Res Microbiol.

[b53] Rastogi G, Tech JJ, Coaker GL, Leveau JHJ (2010). A PCR-based toolbox for the culture-independent quantification of total bacterial abundances in plant environments. J Microbiol Methods.

[b54] Rastogi G, Sbodio A, Tech JJ, Suslow TV, Coaker GL, Leveau JHJ (2012). Leaf microbiota in an agroecosystem: spatiotemporal variation in bacterial community composition on field-grown lettuce. ISME J.

[b55] Redford AJ, Fierer N (2009). Bacterial succession on the leaf surface: a novel system for studying successional dynamics. Microb Ecol.

[b56] Redford AJ, Bowers RM, Knight R, Linhardt Y, Fierer N (2010). The ecology of the phyllosphere: geographic and phylogenetic variability in the distribution of bacteria on tree leaves. Environ Microbiol.

[b57] Reisberg EE, Hildebrandt U, Riederer M, Hentschel U (2012). Phyllosphere bacterial communities of trichome-bearing and trichomeless *Arabidopsis thaliana* leaves. Antonie Van Leeuwenhoek.

[b58] Ritz K (2007). The Plate Debate: cultivable communities have no utility in contemporary environmental microbial ecology. FEMS Microbiol Ecol.

[b59] Ruppel S, Krumbein A, Schreiner M (2008). Composition of the phyllospheric microbial populations on vegetable plants with different glucosinolate and carotenoid compositions. Microb Ecol.

[b60] Saito A, Ikeda S, Hiroshi E, Minamisawa K (2007). Microbial community analysis of the phytosphere using culture-independent methodologies. Microbes Environ.

[b61] Schloss PD, Handelsman J (2005). Metagenomics for studying unculturable microorganisms: cutting the Gordian knot. Genome Biol.

[b62] Siggins A, Gunnigle E, Abram F (2012). Exploring mixed microbial community functioning: recent advances in metaproteomics. FEMS Microbiol Ecol.

[b63] Stiefel P, Zambelli T, Vorholt JA (2013). Isolation of optically targeted single bacteria by application of fluidic force microscopy to aerobic anoxygenic phototrophs from the phyllosphere. Appl Environ Microbiol.

[b64] Takahashi H, Sekiguchi H, Ito T, Sasahara M, Hatanaka N, Ohba A, Hase S, Ando S, Hasegawa H, Takenaka S (2011). Microbial community profiles in intercellular fluid of rice. J Gen Plant Pathol.

[b65] Telias A, White JR, Pahl DM, Ottesen AR, Walsh CS (2011). Bacterial community diversity and variation in spray water sources and the tomato fruit surface. BMC Microbiol.

[b66] Teplitski M, Warriner K, Bartz J, Schneider KR (2011). Untangling metabolic and communication networks: interactions of enterics with phytobacteria and their implications in produce safety. Trends Microbiol.

[b67] Thompson IP, Bailey MJ, Fenlon JS (1993). Quantitative and qualitative seasonal changes in the microbial community from the phyllosphere of sugar beet (*Beta vulgaris*. Plant Soil.

[b68] Tomás-Callejas A, López-Velasco G, Camacho AB, Artés F, Artés-Hernández F, Suslow TV (2011). Survival and distribution of *Escherichia coli* on diverse fresh-cut baby leafy greens under preharvest through postharvest conditions. Int J Food Microbiol.

[b69] Vokou D, Vareli K, Zarali E, Karamanoli K, Constantinidou H-IA, Monokrousos N, Halley JM, Sainis I (2012). Exploring biodiversity in the bacterial community of the Mediterranean phyllosphere and its relationship with airborne bacteria. Microb Ecol.

[b70] Vorholt JA (2012). Microbial life in the phyllosphere. Nat Rev Microbiol.

[b71] Whipps JM, Hand P, Pink D, Bending GD (2008). Phyllosphere microbiology with special reference to diversity and plant genotype. J Appl Microbiol.

[b72] Wilson M, Lindow SE, Colwell RR, Grimes DJ (2000). Viable but nonculturable cells in plant-associated bacterial populations. Nonculturable Microorganisms in the Environment.

[b73] Yashiro E, Spear RN, McManus PS (2011). Culture-dependent and culture-independent assessment of bacteria in the apple phyllosphere. J Appl Microbiol.

[b74] Zhou Y, Qiao X, Li W, Xu J, Wang W, Chen X (2011). Phyllosphere bacterial communities associated with the degradation of acetamiprid in *Phaseolus vulgaris*. Afr J Biotechnol.

